# Deep learning to infer visual acuity from optical coherence tomography in diabetic macular edema

**DOI:** 10.3389/fmed.2022.1008950

**Published:** 2022-10-06

**Authors:** Ting-Yi Lin, Hung-Ruei Chen, Hsin-Yi Huang, Yu-Ier Hsiao, Zih-Kai Kao, Kao-Jung Chang, Tai-Chi Lin, Chang-Hao Yang, Chung-Lan Kao, Po-Yin Chen, Shih-En Huang, Chih-Chien Hsu, Yu-Bai Chou, Ying-Chun Jheng, Shih-Jen Chen, Shih-Hwa Chiou, De-Kuang Hwang

**Affiliations:** ^1^Doctoral Degree Program of Translational Medicine, National Yang Ming Chiao Tung University and Academia Sinica, Taipei, Taiwan; ^2^School of Medicine, National Yang Ming Chiao Tung University, Taipei, Taiwan; ^3^Institute of Clinical Medicine, National Yang Ming Chiao Tung University, Taipei, Taiwan; ^4^Taipei Veterans General Hospital Biostatistics Task Force, Taipei, Taiwan; ^5^Department of Physical Medicine and Rehabilitation, Taipei Veterans General Hospital, Taipei, Taiwan; ^6^Department of Ophthalmology, Taipei Veterans General Hospital, Taipei, Taiwan; ^7^Department of Ophthalmology, National Taiwan University, Taipei, Taiwan; ^8^Department of Physical Therapy and Assistive Technology, National Yang Ming Chiao Tung University, Taipei, Taiwan; ^9^Center for Intelligent Drug Systems and Smart Bio-devices (IDS2B), National Yang Ming Chiao Tung University, Hsinchu, Taiwan; ^10^School of Gerontology and Long-Term Care, College of Nursing, Taipei Medical University, Taipei, Taiwan; ^11^Master Program in Long-Term Care, College of Nursing, Taipei Medical University, Taipei, Taiwan; ^12^International Ph.D. Program in Gerontology and Long-Term Care, College of Nursing, Taipei Medical University, Taipei, Taiwan; ^13^Big Data Center, Department of Medical Research, Taipei Veterans General Hospital, Taipei, Taiwan; ^14^Center for Quality Management, Taipei Veterans General Hospital, Taipei, Taiwan

**Keywords:** treatment response, diabetic macular edema (DME), medical image, visual acuity, deep learning

## Abstract

**Purpose:**

Diabetic macular edema (DME) is one of the leading causes of visual impairment in diabetic retinopathy (DR). Physicians rely on optical coherence tomography (OCT) and baseline visual acuity (VA) to tailor therapeutic regimen. However, best-corrected visual acuity (BCVA) from chart-based examinations may not wholly reflect DME status. Chart-based examinations are subjected findings dependent on the patient’s recognition functions and are often confounded by concurrent corneal, lens, retinal, optic nerve, or extraocular disorders. The ability to infer VA from objective optical coherence tomography (OCT) images provides the predicted VA from objective macular structures directly and a better understanding of diabetic macular health. Deviations from chart-based and artificial intelligence (AI) image-based VA will prompt physicians to assess other ocular abnormalities affecting the patients VA and whether pursuing anti-VEGF treatment will likely yield increment in VA.

**Materials and methods:**

We enrolled a retrospective cohort of 251 DME patients from Big Data Center (BDC) of Taipei Veteran General Hospital (TVGH) from February 2011 and August 2019. A total of 3,920 OCT images, labeled as “visually impaired” or “adequate” according to baseline VA, were grouped into training (2,826), validation (779), and testing cohort (315). We applied confusion matrix and receiver operating characteristic (ROC) curve to evaluate the performance.

**Results:**

We developed an OCT-based convolutional neuronal network (CNN) model that could classify two VA classes by the threshold of 0.50 (decimal notation) with an accuracy of 75.9%, a sensitivity of 78.9%, and an area under the ROC curve of 80.1% on the testing cohort.

**Conclusion:**

This study demonstrated the feasibility of inferring VA from routine objective retinal images.

**Translational relevance:**

Serves as a pilot study to encourage further use of deep learning in deriving functional outcomes and secondary surrogate endpoints for retinal diseases.

## Introduction

The best-corrected visual acuity (BCVA) exam is the most popular test to reflect the condition of the central fovea and the severity of many ocular diseases. Introduced in 1862 by Herman Snellen, the visual chart remained the gold standard for visual acuity (VA) clinical measurement. Visual charts rely on the ability of the patient to identify rows of letters at a fixed distance as each row (line) appears increasingly smaller in size. Although the chart performance depends on the subjective nature of the human response, chances in the correct guessing, or human learning from routine follow-up, the chart remained the basis for VA assessment in clinics and clinical trials. Traditional examinations such as the Early Treatment Diabetic Retinopathy Study (ETDRS) grading scale are usually considered more preferential than other modalities as ETDRS is associated with an escalated risk for vision-threatening retinopathy and serves as a grading scale for retinopathy (1). However, the clinical relevance of the ETDRS grading scale of diabetic retinopathy and other chart-based examinations has been challenged by the difficulty to implement in real-world settings and the technological advances in image acquisition. Thus, the ability to easily derive VA surrogate from routine image modalities provides significant clinical insights throughout the clinical trajectory of macular diseases.

**FIGURE 1:**
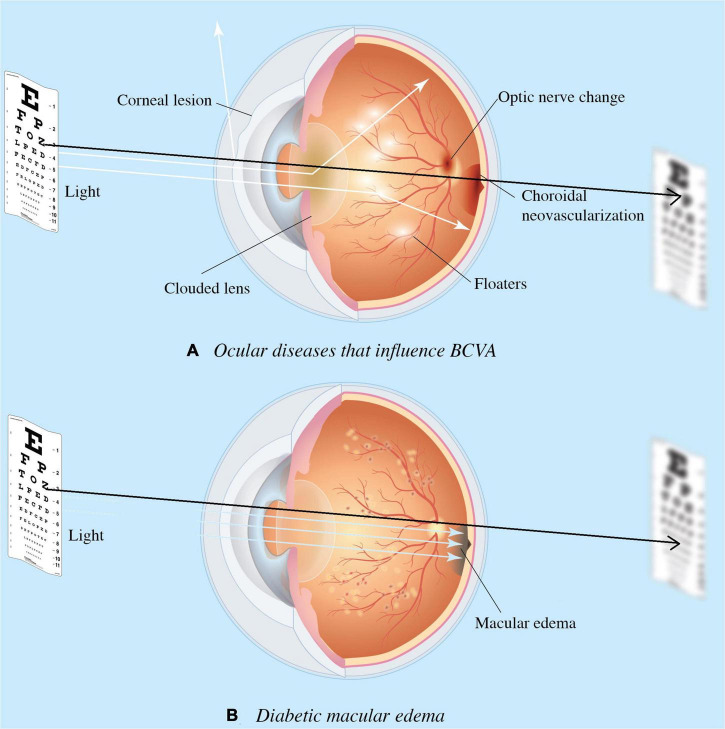
Ocular diseases that influence visual acuity (VA). Diseases impact the visual axis, such as corneal lesions caused by degeneration, clouded lens by cataract, floaters by uveitis, choroidal neovascularization due to age-related macular degeneration (AMD); and optic nerve neuropathy by glaucoma. Such impact obscures diabetic macular edema’s (DME) involvement in the functional outcome of treatment response, and the need for DME treatment. Diagram **(A)** presents concurrent ocular disorders that impact VA measurement, while **(B)** demonstrates the VA directly measures macular health when isolate DME is present. Black arrow denotes the visual axis.

Since the introduction of intravitreal injections (IVI) anti-VEGF, physicians are able to treat exudative macular diseases and recover VA (2–5). In clinical practice, ophthalmologists rely on multiple information, accumulated experience, and intuitive predictions to predict diabetic macular edema (DME) treatment response and whether the treatment is worth pursuing based on an individual’s response (6, 7). In daily clinical practice, clinicians often encounter DME patients with concurrent ocular diseases (Figure 1) . Therefore, traditional VA examinations based on charts may not wholly reflect DME status or be accurately quantified. For this reason, we aimed to provide surrogate VA based on optical coherence tomography (OCT) that depict macular structural health directly.

Optical coherence tomography is routinely used to screen patients with macular disease where the technology depicts the structural retinal health via scans of retinal cross layers (8–10). Besides, the popularity of OCT across medical settings (i.e., optic glass store, non-ophthalmic clinics) makes the utility practical for disease screening and earlier referral. The wealth of information generated via non-invasive retinal scans makes the technology ideal to distinguish baseline status and treatment response (11–14). The ability to infer surrogate VA from OCT and by assisting physicians in detecting OCT-VA and chart-based VA mismatch will allow the physician to derive treatment strategies taking account of concurrent ocular disease to maximize VA recovery.

To evaluate the potential of deep learning in predicting VA outcomes from structural and functional assessments in the early stages of the diagnosis, we built an SD-OCT-based deep learning model using real-world data to infer the VA cut-off value of 0.50, consistent with the minimal requirement for referral by the AAO (15). To our knowledge, this is the first study to implement deep learning in inferring VA from OCT images in DME patients.

## Methods and materials

### Ethical approval and data source

This study was approved by the Institutional Review Board (IRB) of Taipei Veterans General Hospital (TVGH) and written informed consent was signed. This study does not include minors, or minorities. Optical coherence tomography (SD-OCT) B-Scans were selected as the primary input information to establish the computer-assisted visual acuity diagnosis system. All OCT images and subjective, objective, assessment, and plan (SOAP) notes between February 2011 and August 2019 were retrieved from the databank in the big data center (BDC) of TVGH. This dataset consists of de-identified secondary data released for retrospective research purposes. In addition, the OCT images were collected from the patients diagnosed with diabetic macular edema (DME) who sought medical help in the TVGH’s Department of Ophthalmology and received an ophthalmology image inspection using the RTVue XR AngioVue OCT device (Optovue Inc., Fremont, CA, USA).

### Study participants

Patients were enrolled based on the following inclusion criteria: (1) age above 20 years old, (2) diagnosis of diabetes mellitus (I or II), (3) diagnosis of DME with available baseline OCT image and VA, (4) BCVA measured by Snellen chart from 0.05 to 1.50 (decimal), (5) central-involved macular edema defined by the retinal thickness of >250 μm in the central subfield based on Optovue’s automated quantification and the presence of intraretinal fluid (IRF) and subretinal fluid (SRF) seen on SD-OCT, Exclusion criteria were as follows: the presence of cataract or clouded lens, without cataract surgery records. The ocular conditions were obtained from the clinical charts documented by ophthalmologists on the same day when OCT images were taken. In addition, patient charts were reviewed for demographic data, hemoglobin A1C (HbA1C) values, and BCVA.

### Clinical labeling

**FIGURE 2:**
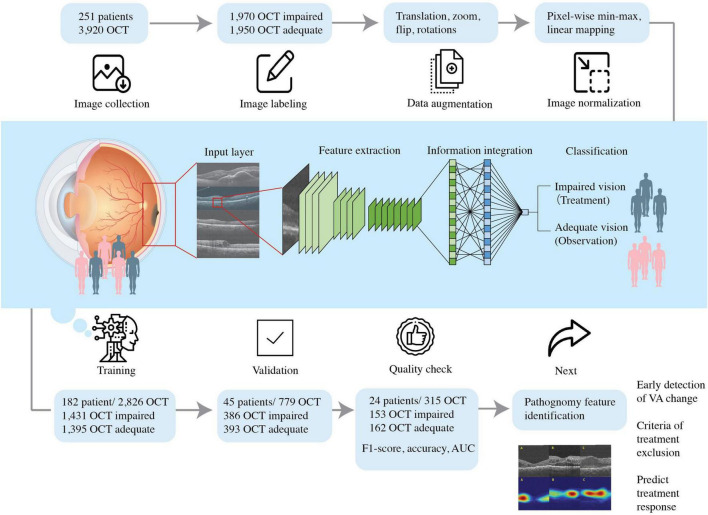
Schematic diagram showing the flow of this study. We included patients diagnosed with DME with the best- BCVA between 0.05 and 1.50 and collected the optical coherence tomography (OCT) dataset. The dataset was labeled accordingly with BCVA obtained and DME OCT features by experienced ophthalmologists. The pre-processed OCT database trained the convolutional neural network, so the artificial intelligence algorithm could predict VA and guide therapeutic strategy.

**FIGURE 3:**
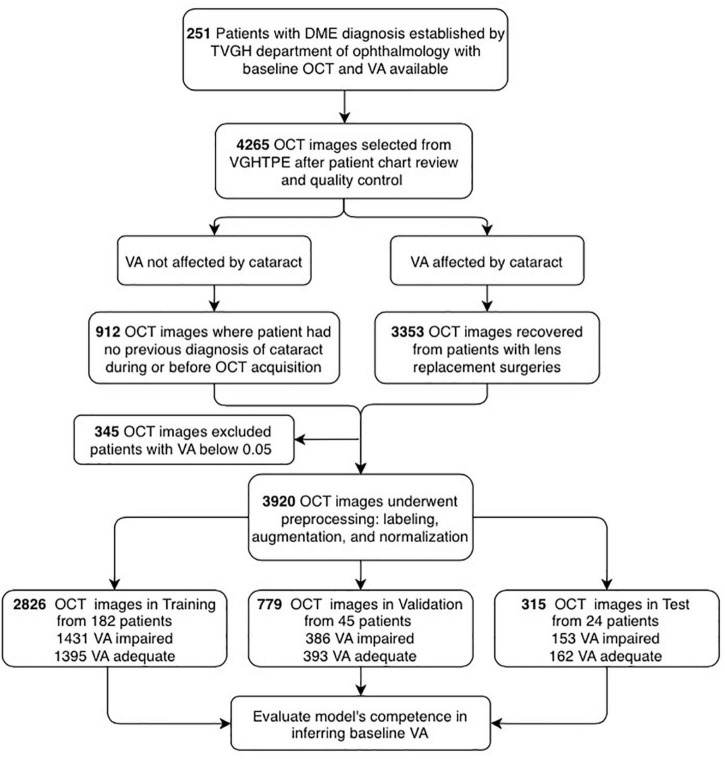
Flowchart showing the selection of optical coherence tomography (OCT) images and their analysis. OCT images and patient clinical information were de-identified secondary data released for retrospective research purposes (*N* = 4,265). The OCT images were collected from the patients diagnosed with DME with clear lens (*N* = 912) or artificial intraocular lens (*N* = 3,353), best-corrected visual acuity (BCVA) measured from 0.05 to 1.5 (decimal), excluding those with lower than 0.05 (*N* = 3,920). Afterimage preprocessing, the dataset was categorized into training (*N* = 2,926), validation (*N* = 779), and test (*N* = 315) for the establishment of the AI platform.

Best-corrected visual acuity of both eyes was measured on the same day when OCT images were acquired in the Department of Ophthalmology, TVGH. Physicians obtained each OCT scan with a ground truth BCVA and documented it on chart review in each visit. We excluded patients with unspecified BCVA or profound visual impairment defined by the International Classification of Diseases, 11th Revision (ICD-11) as BCVA of decimal notation less than 0.05. Our study employed the cut-off value of 0.50, consistent with the minimal requirement for referral by the American Association of Opthalmology (AAO) (15). We defined BCVA values greater than or equal to 0.50 labeled as “adequate” and those less than 0.50 as “impaired” (Figures 2, 3). The same 0.50 thresholds to discriminate against patients with adequate and impaired vision is consistently used in the literature (16–18).

### Datasets and image pre-processing

All participants in this retrospective study were selected based on a comprehensive ophthalmic examination. OCT is accessed via the Big Data Center where reports containing horizontal scan and vertical scan of mid-foveal position is uploaded as PDF reports by Optometrist to the institutions medical image storage PACS (Picture archiving communication system). We cropped the region of interest (ROI) from both vertical and horizontal scans and saved the image in png format (resolution 1960 × 645, bit depth 8) for subsequent model development. The ROI is extractracted from the middle one third of scan areas and downsized them to 224 × 224 pixels resolution by bicubic interpolation. The images were divided into training, validation, and testing groups (Figures 2, 3). First, 70% of the images were incorporated into the training group to train and generate the model parameters. Then, the model’s performance was checked by evaluating an independent validation group (20%). The model that generated the smallest error was designated as the final model. Finally, the test group was composed of the remaining dataset (10%) independent of the training. This group was used to appraise the accuracy rate of the final model. To improve deep learning DL efficiency, we conducted data augmentation by horizontal and vertical translation, zooming, Gaussian blurring of the additional noise, horizontal flipping, and random rotation within 30° translation, zooming, Gaussian blurring of the additional noise, horizontal flipping, and random rotation within 30°. The augmented dataset was used only for training and not validation or testing. The resized or augmented images then underwent pixel-wise min-max normalization, linear mapping of pixel intensities to the range [−1, 1]. We then used the F1-score, accuracy, and area under the curve (AUC) to evaluate the AI model’s performance. F1-score evaluates the test’s accuracy calculated from the test’s precision and recall (sensitivity) (Figure 2).

### Establishing the artificial intelligence models

An efficient recognition algorithm, convolutional neural network (CNN), is frequently used in image processing and pattern recognition (19, 20). We used EfficientNet-B0 deep neural network architecture to classify OCT images in this study (21). Employing transfer learning, we compared EfficientNet-B0 with models of different network architectures, VGG11, VGG16, and ResNet34, which were pre-trained for different tasks, converged them for considerably faster steady value, and reduced training time. Furthermore, the AI models were established using the Google cloud platform with two-core vCPU, 7.5 GB RAM and an NVIDIA Tesla K80 GPU card; the software used was CentOS7 with Keras 2.2.4 and TensorFlow-GPU 1.6.0 for training and validation. Because of the retina’s size and shape variations, a stochastic gradient descent (SGD) algorithm trained the computational layers with a relatively small batch size (32 images). The total training iteration was 310 epochs; the learning rate was le-4 in the first ten epochs, and the learning rate was downgraded to le-5 in the successive epochs. The training for all categories was performed for 310 epochs, and the loss was calculated using the binary cross-entropy loss function.

**FIGURE 4:**
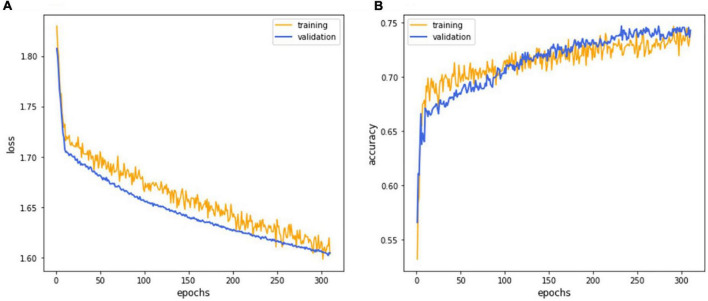
The deep learning model training curve. The CNN model EfficientNet training process revealed that iterations attained lower loss **(A)** and higher accuracy **(B)** as the model underwent successive iterations with the 252nd epoch representing the best performance.

To prevent overestimating and overfitting our model’s performance, we ensured that both the previous train-test split and the subdivision of the training set were done “patient-dependent” to ensure that no images from a single patient could appear in training corresponding validation sets. The final model parameters, listed in Table 1, were selected based on the validation set’s accuracy (Figure 4) and used for the testing set.

**TABLE 1:** The details of the final trained models.

Parameters	Setting
Architecture	EfficientNet
Optimizer	SGD
Loss function	Binary cross-entropy
Learning rate	1e-4 and 1e-5
Batch size	32
Total number of epochs run during training	310

The final model parameters showed the most superior performance where we also compared transfer learning models of the different network architectures, VGG11, VGG16, and ResNet34.

### Final test and clinical evaluation

To evaluate the final AI model’s performance, we used the confusion matrix and the receiver operating characteristic curve (ROC curve) (22, 23). The confusion matrix, comprising four parameters such as true positive (TP), true negative (TN), false positive (FP), and false-negative (FN), was used to evaluate the accuracy, precision, recall (sensitivity), and F1-score. The ROC curve evaluated the false-negative performance with both continuous and ordinal scales (24). Negatives were summarized with a graphical plot of 1-specificity against the sensitivity and the area under the ROC curve (AUC). Attempting to fathom which pathognomy features were critical in associating with BCVA, we used the Grad-CAM technique to visualize the heat map of AI’s recognitions (25–27).

## Results

### Image collection

A total of 259 patients with DME were recruited, and eight patients with visual acuity of decimal notation less than 0.05 were excluded. The participants were mostly over 60 years old, with an average age of 63 years. The ratio of males was 130 (51.8%). While 17.5% of patients had clear lenses, the remaining 82.5% had undergone intraocular lens (IOL) surgery. The database contained 3,920 images. Images from 24 randomly selected patients (9.6% of 251 patients) were preserved as the final test set, and the rest of the images constituted the training and validation sets. A total of 182 and 45 patients have been assigned to training and validation datasets, respectively. Therefore, a total of 1,431 OCT images labeled as “impaired vision” and 1,395 OCT images labeled as “adequate vision” constituted the training set (70% of all enrolled images), the validation dataset (20% of all enrolled images) contained 386 OCT images with “impaired vision” label and 393 OCT images with “adequate vision” label. The test dataset (10% of all enrolled images) was composed of 315 OCT images, which contained 162 images with an “adequate vision” label and 153 images with an “impaired vision label,” as shown in Table 2. Besides, BCVA values of the impaired and adequate groups dataset were similar in each dataset (the visual acuity of the impaired group and adequate group in each dataset was close to 0.22 and 0.68, respectively) (Table 2).

**TABLE 2:** The details of the training, validation, and final test datasets list the numbers of allocated patients and optical coherence tomography (OCT) images and average BCVA values of patients.

Dataset	Training	Validation	Final test
Number of patients	182	45	24
Number of images	2,826 (1,431 impaired, 1,395 normal)	779 (386 impaired, 393 normal)	315 (153 impaireds, 162 normal)
BCVA (SD)	Impaired 0.22 (0.12)	Impaired 0.23 (0.13)	Impaired 0.22 (0.14)
	Normal 0.68 (0.17)	Normal 0.69 (0.20)	Normal 0.66 (0.14)

BCVA, best-corrected visual acuity; SD, standard deviation.

### Model development

The CNN model EfficientNet achieved superior performance during the training process and was selected as the final model for subsequent verifications. The training process’s detailed learning curve revealed that iterations attained lower loss and higher accuracy as the model underwent successive iterations (Figure 4). Finally, the validation accuracy curve achieved a testable level, and the training accuracy was higher than the validation accuracy, which meant that the training process was finished. The 232*^nd^* epoch represented the best performance of the validation accuracy (76.1%). Hence, this trained AI model has been selected as the final model to execute the final test.

### The final test of the trained artificial intelligence model

**FIGURE 5:**
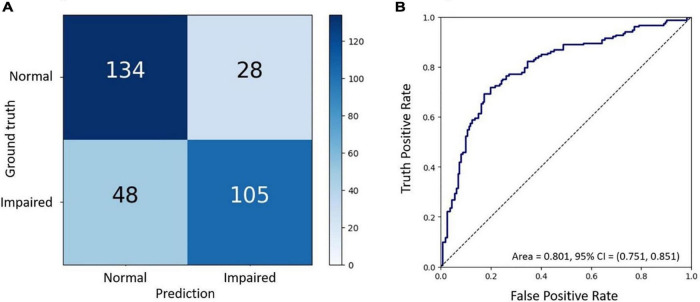
The final test of the trained AI model. **(A)** Confusion matrix demonstrating the accuracy of prediction of two visual acuity classes based on the validation dataset of OCT images. **(B)** Receiver operating characteristic (ROC) curve showing the accuracy of prediction with the area under the curve (AUC) = 0.801.

**FIGURE 6:**
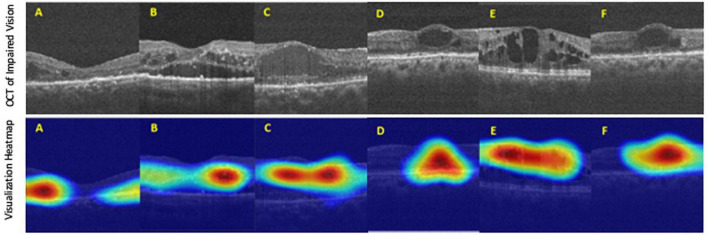
The heat map visualization of representative six **(A–F)** OCT images recognized by our AI model as predictors of best-corrected visual acuity (BCVA)-defined impaired vision. *Top panel*: original input images from the final test dataset. *Bottom panel*: heat map visualization of areas used by our AI model to discriminate between BCVA classes.

Finally, the final trained AI model was verified by the final test dataset to evaluate its realistic performance. The test dataset contained 162 images with the “adequate vision” label and 153 images with the “impaired vision” label. Our AI model’s accuracy, precision, recall, and F1-score were 75.9, 68.6, 78.9, and 73.4%, respectively (Figure 5A). As was calculated from the receiver operator characteristic (ROC) curve, the area under the curve (AUC) was 0.801, with the confidence interval (CI) from 0.751 to 0.851 (Figure 5B). Furthermore, we applied heat map visualization to identify OCT image areas recognized by the AI to discriminate between BCVA classes (Figure 6). The heat maps highlighted a more extensive area covering nearly the entire retinal layer instead of specific smaller lesions in some cases. The more extensive coverage of heat maps identified by AI to be critical for the determination of BCVA could be related to the multiple microstructural changes and the thickness of the retina.

## Discussion

Artificial intelligence in DR screening and referral decisions has achieved clinical reality. The first FDA-approved AI system (2018), IDx-DR (Iowa, USA), can analyze the digital fundus photograph (FP) in DR screening to provide referral suggestions (23). Apart from using the FP-based AI model, researchers have also developed an OCT-based algorithm, Notal OCT analyzer (NOATM, Notal Vision, Israel), which uses deep learning algorithms to detect the retinal fluid in AMD patients (24). However, up-to-date, in our literature review, there is no study focusing on an AI-based model to evaluate visual acuity in DR nor DME. Thus, our study developed an OCT-based AI model that could infer the binary VA status separated by the threshold of 0.5 (decimal notation) and attained an accuracy of 75.9%, a sensitivity of 78.9% with AUC = 80.1% based on OCT images only (Figure 4). Furthermore, to verify that our deep learning model indeed analyzed the exacted structural features of DME, we applied the heat map visualization to graphically show the different weighted values in pixel matrices of OCT images (Figure 6).

While visual acuity is a clinical measurement of changes in visual function as a primary endpoint, FDA recommends that retinal imaging technologies help determine anatomic markers for clinical progression of the disease. With the advent of imaging technologies such as the color fundus, angiography, and OCT, clinicians can observe the structural health of the neurosensory retina and generate new endpoints not previously accessible. For example, current technologies can identify onset, or progressions before symptom occurrence, leading to smaller marginal changes for earlier intervention and better visual outcomes. The use of OCT images was therefore incorporated into the work routine of the ophthalmologist to quantify the structural changes in individual patients’ retinal pathological and topographic profiles (28). In addition, the ease of use and adoption into routine clinical practice makes the technology powerful to derive surrogate endpoints that change along with clinical endpoints and represent the disease status.

There are several limitations to our study. Rather than inferring the continuous VA variable, we only employed a binary classification of “impaired” and “normal” VA. Some may argue that the grade of impairment is essential as we may evaluate whether the patient is close to the treatment threshold or far away. Linear regression was not performed as we face small sample size that does not follow the assumption of normality, constant variance, and independent sampling, to construct a robust model in predicting visual function status at the decimal-level. Besides, our small sample size coupled with real-world heterogeneity caused our standard deviation of VA relatively large and BCVA measurements in the clinic may not be recorded as vigorously in controlled trials with EDTRS logmar standard. Our deep learning model may assist in evidence-based assistance to the physician, alleviating their burden in determining those with impaired vision (less than 0.5 baseline VA). Moreover, we only excluded patients with cataract diagnoses without cataract surgery. To achieve a better yield, we ideally have to impose exclusion criteria such as (1) prior history of choroidal neovascularization due to AMD, retinal vein occlusion, uveitis, or any other inflammatory disease, (2) presence of cataract or clouded lens, (3) glaucoma or any other neuropathy, (4) epiretinal membrane, vitreomacular traction disease, or any other maculopathy, and (5) corneal disease or degeneration. By only excluding cataracts, we obtain broader inclusion criteria that allow this AI model to closely imitate real-world settings and be expanded to accommodate most DME patients. Our AI model may be extended to serve a wider population by not excluding patients who underwent previous treatment and can be used for screening, referral, and monitoring. Finally, our model is constructed with horizontal and vertical scans of the mid-foveal position and not OCT volume. Therefore, we cannot analyze the concordance of the binary outcome of several OCT slices of the same patient and quantify their contradicting outcomes.

In the future, inferring VA based on imaging may be considered as quasi-functional surrogate endpoints for interventional clinical trials. By doing so, clinical trials can enroll a larger set of patients that resemble those in the real world and provide treatment recommendations that can be implemented in the clinic (29–31). Furthermore, DME results in loss of visual function long before visual acuity is impaired as central acuity is not always affected. Herein, only a subgroup of DME fits the standard. Clinically significant macular edema (CSME) is defined as a lesion within 500 μm of the foveal center and center involved macular edema as central subfield retinal thickness of >250 μm in central 1 mm ETDRS grid (foveal thickness). Much macular retinal health recovery is not reflected in visual acuity. Visual acuity measured by visual charts (EDTRS, Snellen test) measures the photopic function of the central retina and is not reflective or sensitive to gain of retinal health or therapeutic benefits. Therefore, it is proposed that patient-reported outcome measures assess impairment of visual function in more detail. Redefine investigation of treatment effects superior to standard visual acuity testing without the need for extensive psychophysical examination. The European Medicines Agency (EMA) and FDA now demand the employment of patient-reported outcome measure (PROM) as functional endpoints in clinical trials (NEI-VFQ-25) are now routinely used as a valid and reliable measure of patients’ vision-related quality of life. However, these tests are time-consuming, demanding for the elderly patient, and present significant inter-interpreter variability. In addition, rather than inferring function in a cross-sectional time manner for baseline VA, another interesting aspect is to predict VA in the future – what are the estimated letter gains after IVI-VEGF for my disease status? These algorithms inform patients about treatment prognosis and give patients the power to self-assess the cost-benefit of pursuing the IVI-VEGF. Overall, sensitive and robust outcome measures of retinal function are pivotal for measuring the clinical trial primary endpoint of VA and reinforce patient autonomy in the decision-making process.

## Conclusion

This study built an OCT-based deep learning model that inferred VA status based on OCT and was correlated with the concurrent BCVA measured by standard visual charts. We achieved an accuracy of 75.9%, sensitivity of 78.9%, and a ROC AUC of 80.1%. This demonstrated the feasibility of predicting the functional outcome VA from routine ophthalmic images and served as a pilot study to develop further surrogate markers that can better represent the disease.

## Data availability statement

The datasets presented in this article are not readily available due to legal, ethical and privacy restrictions of hospital data. Further inquiries can be directed to the corresponding author.

## Ethics statement

The studies involving human participants were reviewed and approved by Institutional Review Board (IRB) of Taipei Veterans General Hospital (TVGH). Written informed consent for participation was not required for this study in accordance with the national legislation and the institutional requirements.

## Author contributions

Y-CJ, Y-BC, S-JC, and D-KH contributed to the conception and design of the study. T-CL, C-HY, C-LK, P-YC, S-EH, S-HC, C-CH, and Z-KK organized the database. T-YL, H-RC, and H-YH performed the statistical analysis. T-YL and Y-IH wrote the first draft of the manuscript. K-JC wrote sections of the manuscript. All authors contributed to the manuscript revision, read, and approved the submitted version.
